# A Novel Intronic Mutation in MBD5 Results in Autosomal Dominant Intellectual Disability Type 1 due to Abnormal Splicing

**DOI:** 10.1002/mgg3.70121

**Published:** 2025-07-15

**Authors:** Heng Jiang, Jingjing Mou, Qiwei Zhao, Long Ding, Yu Wang, Xiaohong Guo, Guohua Yang

**Affiliations:** ^1^ Department of Medical Genetics School of Basic Medical Science, Wuhan University Wuhan People's Republic of China; ^2^ Department of Radiation Oncology Hubei Cancer Hospital, Tongji Medical College, Huazhong University of Science and Technology Wuhan Hubei People's Republic of China; ^3^ Department of Basic Medicine Hubei University of Chinese Medicine Wuhan People's Republic of China; ^4^ Hubei Provincial Key Laboratory of Developmentally Originated Disease Wuhan People's Republic of China

**Keywords:** *MBD5*, MRD1, mRNA splicing abnormalities, neurodevelopmental disorder

## Abstract

**Background:**

We identified a novel variant in *MBD5* located within intron 6: c.114‐13A>G (NM_018328.5) in a family with a patient presenting intellectual disability. This variant is hypothesized to be the etiological agent underlying the patient's condition.

**Methods:**

We conducted an analysis of mRNA splicing within the patient and their relatives' blood samples via reverse transcription polymerase chain reaction (RT‐PCR) to assess intronic mRNA splicing. Additionally, we employed a minigene vector construction approach to verify in vitro the splicing of mRNA containing the mutated fragment. Protein structure prediction analysis of the aberrant mRNA was performed using PyMOL software.

**Results:**

The patient harbors a novel mutation in the *MBD5* gene: c.114‐13A>G. Analysis of the patient's blood sample revealed the presence of aberrantly sized mRNA molecules. Utilizing a minigene approach, we discovered that this mutation results in the generation of two types of abnormally sized mRNAs. The first type of abnormal splicing leads to a 12‐base pair retention at the 3′ end of intron 6, and the second type of abnormal splicing causes exon 7 skipping.

**Conclusion:**

In accordance with the “Standards and Guidelines for the Interpretation of Sequence Variants” established by the American College of Medical Genetics and Genomics (ACMG), the novel mutation c.114‐13A>G in the *MBD5* gene meets the criteria for PS2 (the variant is de novo and not inherited from either parent) and PS3 (the variant affects mRNA splicing, resulting in aberrant transcripts). We propose that the c.114‐13A>G variant, located within intron 6 of the *MBD5* gene, is pathogenic. This discovery not only expands the repository of pathogenic variants for *MBD5* but also provides additional insights into intronic mutations of the *MBD5* gene, thereby offering significant information for the genetic diagnosis of Autosomal Dominant Intellectual Disability Type 1.

## Graphical abstract

The novel intronic potentially pathogenic mutation in the MBD5 gene results in aberrant splicing of mRNA within the body. The protein transcribed from this abnormal mRNA undergoes changes in its spatial structure, with alterations in the electrostatic potential on the protein surface. This newly identified mutation may be the cause of the patient's disease, providing a new pathogenic mutation site for the MBD5 gene in clinical research and expanding the mutation database for the MBD5 gene.

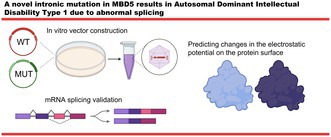

## 1 Introduction

Autosomal Dominant Intellectual Disability Type 1 (MIM#156200), also known as Mental Retardation, Autosomal Dominant 1 (MRD1), is a prototypical neurodevelopmental disorder characterized by intellectual disability, developmental delays, and potential physical anomalies, including distinct facial features. The pathogenesis of MRD1 is influenced by several factors, including abnormal methylation, neurodevelopmental impacts, and metabolic abnormalities. Recent advancements in molecular genetics have shed light on the *MBD5* gene (OMIM:611472), offering a new perspective for genetic studies related to MRD1.

MRD1 is closely associated with DNA methylation, a process involving DNA Methyltransferases and the Methyl‐CpG‐Binding Domain (MBD) protein family. Proteins belonging to the MBD family play a crucial role in regulating the transcriptional state of the epigenome by directly recognizing and binding to methylated CpG sites (Gujar et al. 2015; Du et al. 2019). The MBD protein family includes *MBD1, MBD2, MBD3, MBD4, MBD5*, among others. Researchers have conducted functional validations of MBD family proteins in mouse models, leading to the conclusion that these proteins significantly influence DNA methylation (Wade 2001).

The *MBD5* gene, located in the q23.1 region of chromosome 2 in the human genome, is essential for nervous system development and is linked to intellectual disability. This gene is also referred to by its aliases MRD1, DEL2Q23.1, and C2DELq23.1. The *MBD5* gene comprises 15 exons, of which 10 contribute to the coding region, and it is known to have two isoforms. Isoform 1, encoded by exons 6–15, consists of 1448 amino acids and contains two functional domains: an MBD and a Pro‐Trp‐Trp‐Pro (PWWP) domain. In contrast, Isoform 2, which is encoded by exons 6–9 and retains intron 9, results in a protein of 851 amino acids. This isoform also features an MBD domain but lacks the PWWP domain (Mullegama and Elsea 2016). Isoform 1 is expressed across all tissues, with particularly high levels observed in the brain and testes. In contrast, isoform 2 is also present in all tissues but demonstrates especially elevated expression levels in the brain and ovaries (Hamdan et al. 2014). Functional studies indicate that *MBD5* may play a role in the formation or function of heterochromatin (Laget et al. 2010). Haploinsufficiency of the *MBD5* gene can result in syndromes characterized by microcephaly, intellectual disability, severe speech disorders, and epileptic seizures.

Jing et al. (2020) identified five patients with mutations in the *MBD5* gene, specifically c.300C>A, c.1775delA, c.1759C>T, c.150_151del, and c.113+1G>C. These patients presented with comparable clinical features, which included varying degrees of epilepsy and language disorders.

Tadros et al. (2017) identified three mutations in the *MBD5* gene across different families, with parents in two of these families exhibiting the pathogenic gene in a mosaic form. Some patients within these families presented with developmental delays and facial dysmorphism, while others experienced psychological disorders, including autism. These findings suggest that, in the context of Autosomal Dominant Intellectual Disability Type 1, it is essential to consider not only the primary characteristics, such as language disorders and epilepsy, previously documented in the literature, but also the psychological abnormalities associated with *MBD5* gene mutations. Such issues may be overlooked by physicians during clinical diagnosis.

Le and Ha (2007) documented a case involving an intronic mutation in the *MBD5* gene, specifically located at the acceptor splicing site of intron 6, c.217‐1G>C (NM_001378120.1). This mutation was associated with clinical phenotypes including intellectual disability, autism, and epilepsy in the affected patient. Genetic analysis, in conjunction with the patient's phenotypic presentation, confirmed the pathogenic nature of the intronic mutation.

Tang et al. (2023) developed a mouse model of epilepsy and observed an increased expression of *MBD5* in the brain tissue of the epileptic mice. They further validated the distribution and subcellular localization of *MBD5* through immunofluorescence techniques, demonstrating that *MBD5* is primarily localized in the cytoplasm and nucleus of neurons. Tsuboyama et al. (2022) characterized *MBD5* and *MBD6* as essential regulators of the BAP1 complex, which is crucial for maintaining its transcriptional landscape. Their findings also highlight the therapeutic potential of targeting *MBD5* and *MBD6* in BAP1‐dependent human cancers.

Ichino et al. (2021) examined the downstream mechanisms of DNA methylation involved in the transcriptional repression and gene silencing of genes and transposons in eukaryotes. Their study revealed that in *Arabidopsis thaliana* , the methyl‐CpG‐binding domain proteins *MBD5* and *MBD6* are recruited to chromatin through the recognition of CG methylation. These proteins redundantly repress the expression of a subset of genes and transposons without altering DNA methylation levels.

Bhatia et al. (2022) investigated *MBD5* haploinsufficiency and identified a novel deletion mutation in the *MBD5* gene, specifically c.728delC (NM_018328.5). Their family analysis of individuals with heterozygous loss‐of‐function mutations in *MBD5* revealed that all three affected family members displayed varying degrees of developmental delays, behavioral disorders, and language impairments.

We identified a novel mutation in the *MBD5* gene, specifically c.114‐13A>G (NM_018328.5), located within intron 6 of a family. To assess whether this mutation affects mRNA splicing, we conducted splicing validation analyses both in vivo and in vitro. The results consistently demonstrated that the mutation led to the production of aberrant transcripts of the *MBD5* gene, which may result in abnormal protein sequences and contribute to the clinical phenotype of MRD1 observed in the affected patients.

## 2 Method and Materials

### 2.1 Editorial Policies and Ethical Considerations

This study was approved by the Wuhan University Ethics Committee for Life and Medical Sciences, and informed written consent was obtained from all patients. We obtained informed consent from the patient's parents. Participants have provided written consent for the publication of clinical information and image data in this study. This study adheres to the Declaration of Helsinki, and the human data and materials used are in compliance with the Declaration of Helsinki.

### 2.2 Patient Recruitment and Sample Collection

This study recruited an independent MRD1 family, from which peripheral blood samples were collected from the parents and younger brother for whole‐exome sequencing (WES), and from the proband for whole‐genome sequencing (WGS). Additionally, the *MBD5* gene (GenBank ID: NM_018328.5) was amplified from the proband, their parents, and younger brother to validate the findings through Sanger sequencing. Informed consent was obtained from all participants.

### 2.3 DNA Sequencing and Mutation Analysis

The study employed a blood DNA mini kit for the extraction of genomic DNA from peripheral blood samples. Subsequently, high‐throughput sequencing was utilized to analyze the genomic sequences of the patient. This process involved extracting DNA from the blood samples, constructing libraries, and employing high‐throughput next‐generation sequencing to sequence the entirety of the human genome. Additionally, a bioinformatics analysis was performed on the mutation sites to assess the mutations and predict their potential impact on mRNA.

### 2.4 In Vivo Splicing Analysis of the *MBD5* Gene

Total RNA was extracted from blood samples utilizing the Blood Total RNA Extraction Kit (TR121‐10, GENSTONE BIOTECH). Following this, mRNA was reverse transcribed into complementary DNA (cDNA) using the PrimeScript FAST RT Reagent Kit with gDNA Eraser (RR092A, TaKaRa). All PCR primers and base sequences used in this experimental process are specifically presented in Table S1. PCR amplification of the cDNA was conducted with primers MBD5‐F1 and MBD5‐R1. The resultant product served as a DNA template for subsequent PCR amplification using primers MBD5‐F2 and MBD5‐R2. The amplified product was then analyzed through DNA gel electrophoresis to assess the in vivo splicing pattern of the *MBD5* gene. After purifying the bands, TA cloning was performed, followed by Sanger sequencing.

### 2.5 *MBD5* Gene Minigene Vector Construction

Using nested polymerase chain reaction (PCR) as a template, we successfully obtained a wild‐type fragment of the *MBD5* gene from genomic DNA. For the in vitro minigene experiments on the *MBD5* gene of the patient, two distinct sets of vectors, pcMINI and pcMINI‐c, were chosen.

Two pairs of nested primers, 2530‐MBD5‐F and 5669‐MBD5‐R, as well as 2811‐MBD5‐F and 5490‐MBD5‐R, were designed for nested PCR utilizing normal human genomic DNA (gDNA) as the template. The product from the second round of nested PCR served as the template to amplify the wild‐type fragment of pcMINI, which is 1327 bp in length, using the primers pcMINI‐MBD5‐KpnI‐F and pcMINI‐MBD5‐XhoI‐R. Similarly, the left half of the mutant fragment of pcMINI, measuring 782 bp, was amplified with primers pcMINI‐MBD5‐KpnI‐F and MBD5‐mut‐R, using the product of the second round of nested PCR as the template. The right half of the mutant fragment, 586 bp in length, was amplified with primers pcMINI‐MBD5‐XhoI‐R and MBD5‐mut‐F, again utilizing the product of the second round of nested PCR as the template. Finally, a 1:1 mixture of the left and right halves of the mutant fragment served as the template to amplify the complete mutant fragment of pcMINI, which is also 1327 bp in length, using the primers pcMINI‐MBD5‐KpnI‐F and pcMINI‐MBD5‐XhoI‐R.

The wild‐type fragment of pcMINI‐C, measuring 2001 bp, was amplified using the product of the second round of nested PCR as the template, with primers pcMINI‐C‐MBD5‐KpnI‐F and pcMINI‐C‐MBD5‐XhoI‐R. In parallel, the left half of the mutant fragment of pcMINI‐C, which is 659 bp in length, was amplified using the same PCR product as a template, employing primers pcMINI‐C‐MBD5‐KpnI‐F and MBD5‐mut‐R. The right half of the mutant fragment, measuring 1383 bp, was also amplified using the product from the second round of nested PCR as the template, utilizing primers pcMINI‐C‐MBD5‐XhoI‐R and MBD5‐mut‐F. Finally, a 1:1 mixture of the left and right halves of the mutant fragment was used as the template to amplify the complete mutant fragment of pcMINI‐C, which is 2001 bp in length, employing the same primers, pcMINI‐C‐MBD5‐KpnI‐F and pcMINI‐C‐MBD5‐XhoI‐R.

Four recombinant vectors were transfected into HeLa and 293 T cell lines. After a 48‐h transfection period, eight samples were collected for RNA extraction, which was subsequently reverse transcribed into complementary DNA (cDNA). Following this, DNA gel electrophoresis was conducted to examine the band patterns and analyze the outcomes of mRNA splicing.

### 2.6 Cell Transfection and Transcriptional Analysis

293 T and HeLa cells were cultured in DMEM medium supplemented with 10% fetal bovine serum. The constructed wild‐type and mutant eukaryotic recombinant expression vectors were transiently transfected into these cells according to the Lipo2000 liposome protocol. Cell samples were collected 48 h post‐transfection of the wild‐type and mutant vectors. Total RNA was extracted using the Trizol method, and its concentration was measured following DNA digestion. Equal amounts of RNA were utilized for cDNA reverse transcription, and RT‐PCR was conducted to assess mRNA splicing.

## 3 Results

### 3.1 Pedigree of the Proband and Clinical Phenotypes

**FIGURE 1:**
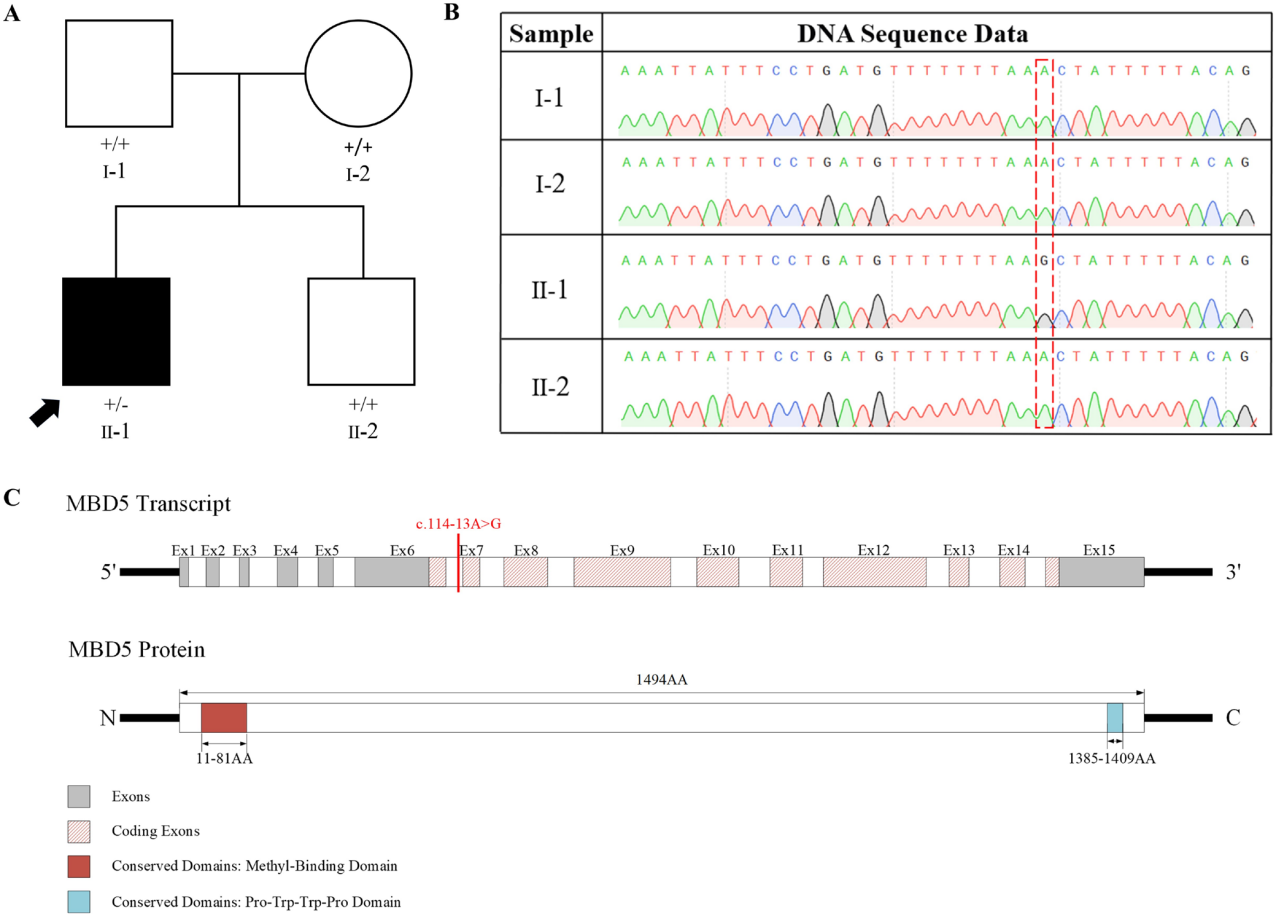
Pedigree of the proband and protein functional domains. (A) Pedigree of the patient; (B) Sanger sequencing results of the family; (C) The corresponding position of the mutation in the gene exon and the protein functional domains of *MBD5*.

We identified a family affected by MRD1 (Figure 1A). The child was conceived naturally, and there were no significant events reported during the early stages of pregnancy. At 32 weeks of gestation, fetal monitoring revealed abnormalities. The child was delivered via cesarean section at 36 weeks and 4 days of gestation on November 2, 2021. Following birth, the child was diagnosed with a cow's milk protein allergy and was subsequently fed a specialized formula. The primary manifestations observed include seizures, an abnormal electroencephalogram (EEG), and developmental delays. At the age of 3 years and 11 months, the child had already been diagnosed with mild developmental delay at 1 year and 6 months, and mild developmental defect at 2 years and 8 months. Current developmental challenges include language function impairment, cognitive deficits such as difficulty distinguishing size and color, lack of self‐care skills in toileting, and poor concentration. The child's epilepsy is being managed with Depakine and sodium valproate, although occasional grand mal seizures continue to occur. Additionally, the child underwent adenoidectomy at 2.5 years of age and exhibits occasional drooling.

Through WES and subsequent validation of the candidate gene *MBD5* via Sanger sequencing, we identified a novel intronic mutation, c.114‐13A>G (NM_018328.5), in an independent family. Notably, the proband's father, mother, and younger brother did not exhibit this genetic mutation, suggesting that it is a de novo mutation.

### 3.2 Results of Sanger Sequencing Analysis in the Family

The proband, designated as individual II‐1, possesses the intronic mutation c.114‐13A>G (NM_018328.5) in the *MBD5* gene. Genomic DNA was extracted from the peripheral blood samples of the patient, their parents, and their younger brother. High‐throughput sequencing analysis revealed that neither the parents nor the younger brother carried this mutation, thereby confirming it as a de novo mutation (Figure 1B).

### 3.3 Bioinformatics Analysis of the Impact of the Mutation on the *MBD5* Gene

The *MBD5* gene is situated in the q23.1 region of human chromosome 2 and spans a total of 495,881 base pairs, consisting of 15 exons. The coding region encompasses 54,183 base pairs, which include 10 exons and two major functional domains (Figure 1C), primarily localized in the nucleus. This gene encodes a member of the MBD family, where the MBD consists of approximately 70 amino acid residues, representing the minimal region necessary for MBD to selectively bind to methylated DNA. In addition to the MBD domain, the protein also features a PWWP domain (proline‐tryptophan‐tryptophan‐proline motif), comprising 100–150 amino acids, which is prevalent in various proteins associated with cell division, growth, and differentiation. Mutations in the *MBD5* gene are linked to an autosomal dominant cognitive disorder. Furthermore, haploinsufficiency of this gene may result in syndromes characterized by microcephaly, intellectual disability, severe speech disorders, and epilepsy.

The mutation c.114‐13A>G is situated in intron 6, where the original adenine (A) is substituted by guanine (G). This alteration results in the formation of an abnormal splicing site, leading to aberrant mRNA splicing. To assess the potential impact of this mutation, multiple bioinformatics databases were employed. Algorithms such as SpliceAI Lookup, NNSPLICE version 0.9 (January 1997), and RDDC all indicated that the mutation could influence splicing. Specifically, SpliceAI Lookup predicted the loss of the original acceptor site following the mutation, reflected by a decrease in confidence score to 0.68, while a new acceptor site was identified near the mutation site, with a confidence score of 0.18. These findings suggest that the mutation adversely affects splicing. NNSPLICE version 0.9 corroborated this by concluding that a new acceptor site is generated near the mutation site post‐alteration, further indicating an impact on splicing. RDDC presented three distinct predictions: one indicated the retention of a 12 bp intron following the mutation; another suggested that splicing remained consistent between the mutant and wild‐type; and the third predicted a 103 bp deletion in the mRNA, leading to the skipping of exon 7.

### 3.4 Analysis of the In Vivo Transcriptional Impact of the Mutation on mRNA

**FIGURE 2:**
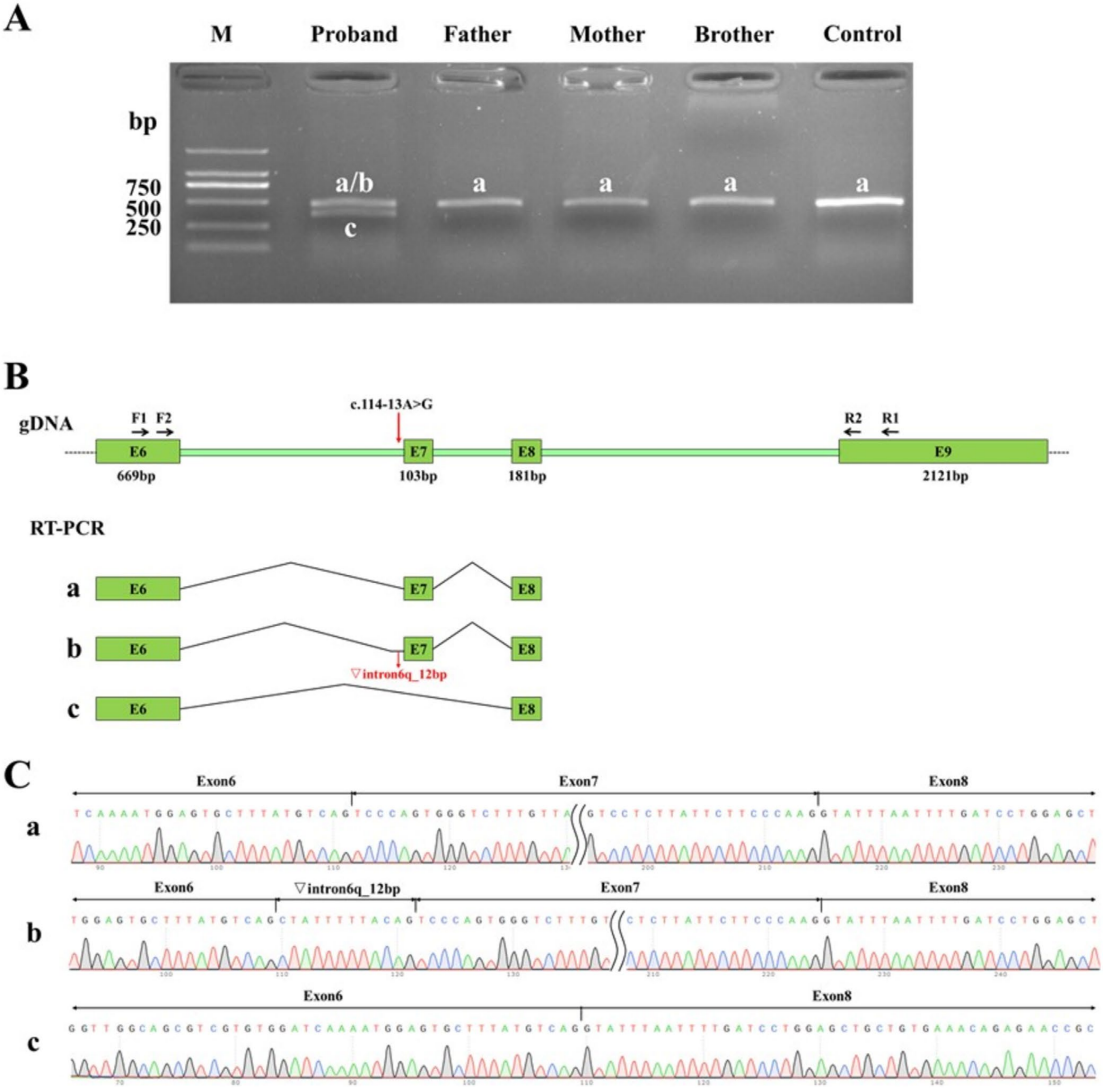
The in vivo mRNA splicing results of the *MBD5* gene. (A) Agarose gel electrophoresis of RT‐PCR products, with bands named in descending order of size as band b, band a, and band c; (B) Schematic diagram of primer design and splicing, with the red arrow indicating the mutation site; (C) Sequencing results corresponding to splicing bands a, b, and c.

T Total RNA was extracted from the blood samples of the patient's parents, younger brother, and the patient. Following reverse transcription to synthesize complementary DNA (cDNA), DNA gel electrophoresis was conducted. The resulting distinct DNA bands were purified and subsequently subjected to TA cloning, leading to the findings illustrated in Figure 2A.

Sequencing analyses revealed a splicing band in normal individuals that corresponded to the expected size of normal splicing (461 bp), designated as band a. In contrast, the proband exhibited three distinct splicing scenarios, with the bands labeled in descending order of size as bands b, a, and c. Notably, a distinct abnormal splicing transcript, referred to as band c, was observed in the proband, characterized by a splicing pattern of exon 6 (669 bp) − exon 8 (181 bp). Band b in the proband represented an abnormal splicing event, exhibiting a 12 bp retention on the right side of intron 6, resulting in a splicing pattern of exon 6 (669 bp) − ▽intron 6 (12 bp) − exon 7 (103 bp) − exon 8 (181 bp). The mutation identified in the proband was de novo and not inherited. In the mRNA of the proband's father, mother, younger brother, and unrelated normal individuals, normal splicing was observed without any abnormalities. We hypothesize that the abnormal splicing of mRNA in the patient is the underlying cause of the disease. The expression of abnormally spliced mRNA leads to the production of proteins with structural and functional defects, ultimately resulting in the clinical phenotype of Autosomal Dominant Intellectual Disability Type 1. Sanger sequencing of the different‐length transcripts confirmed that the abnormal transcripts in the proband aligned with previous predictions (Figure 2C).

### 3.5 *MBD5* Gene Minigene Result Analysis

**FIGURE 3:**
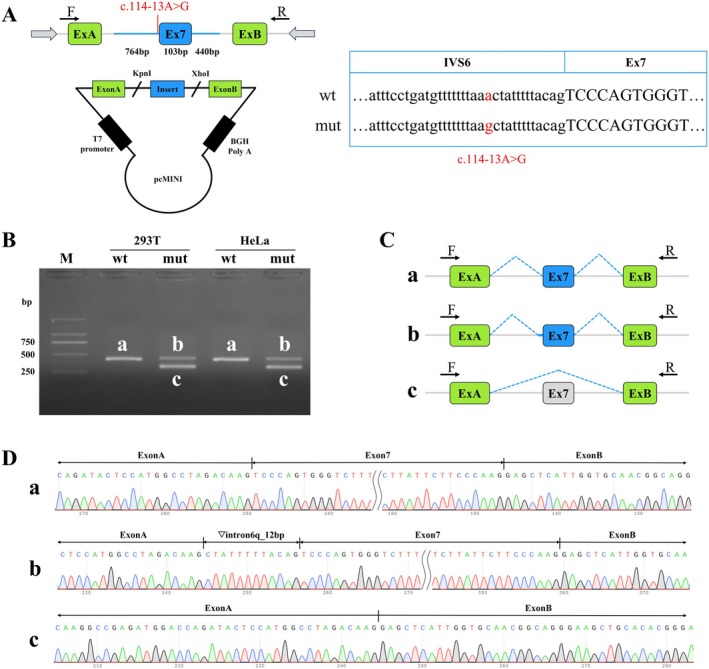
The results of the pcMINI vector for the *MBD5* gene. (A) Schematic diagram of the pcMINI vector construction; (B) Agarose gel electrophoresis of RT‐PCR transcription analysis; bands labeled as a, b, and c in HeLa and 293 T cells; (C) Schematic of splicing for different bands; (D) Sanger sequencing results for different bands.

To ensure the accuracy of the results, the experiment was conducted in triplicate. The expression vectors utilized were from the pcMINI and pcMINI‐C series. Wild‐type and mutant gene fragments were inserted into these vectors, respectively (Figure 3A). Two sets of recombinant vectors were then transfected into 293 T and HeLa cell lines. Following a 48‐h incubation period post‐transfection, samples were collected for RNA extraction, and reverse transcription PCR (RT‐PCR) was performed to analyze the mRNA transcription results for the different vectors.

In the pcMINI vector, a segment comprising part of intron 6 (764 bp), exon 7 (103 bp), and part of intron 7 (440 bp) was inserted (Figure 3A). This vector includes a universal exon A, intron A, multiple cloning site (MCS), intron B, and exon B. Following transfection into cells, the splicing pattern of exon A, exon 7, and exon B was analyzed to identify any abnormalities. The results are presented in Figure 3. In both HeLa and 293 T cells, the wild‐type *MBD5* gene transcript appeared as a single band, corresponding to the expected size of 414 bp, designated as band a. This band was purified from both cell lines and subsequently subjected to Sanger sequencing. In contrast, the mutant type displayed two bands in HeLa and 293 T cells, with the larger band designated as band b and the smaller band as band c (Figure 3B). The mutant bands from both cell lines were purified, TA cloned, and then subjected to Sanger sequencing. The analysis revealed that the wild‐type band a represented a normally spliced transcript, exhibiting a splicing pattern of exon A (192 bp), exon 7 (103 bp), and exon B (57 bp). Conversely, the mutant band b was identified as an abnormally spliced transcript, characterized by a 12 bp retention on the right side of intron 6, resulting in a splicing pattern of exon A (192 bp), intron 6 (12 bp), exon 7 (103 bp), and exon B (57 bp). The mutant band c was also an abnormally spliced transcript, which exhibited exon 7 skipping, leading to a splicing pattern of exon A (192 bp) and exon B (57 bp) (Figure 3D). The experimental results obtained with the pcMINI‐C vector were consistent with those observed for the pcMINI vector, as further demonstrated in Supporting Information S1.

The results of the in vitro minigene experiments indicate that the mutation c.114‐13A>G disrupts the normal splicing of the gene's mRNA, with detection results from both the pcMINI and pcMINI‐C vectors corroborating this finding. Following the mutation, two abnormal transcripts are produced: one exhibits a 12 bp retention on the right side of intron 6, with the inserted sequence being CTATTTTTACAG, while the other involves an exon 7 skipping event. At the cDNA and protein levels, the 12 bp retention manifests as c.113_114insCTATTTTTACAG p.Ser38_Pro39insTyrPheTyrSer. Notably, this retention does not alter the subsequent reading frame; rather, it results in the insertion of four amino acids into the protein, potentially yielding a protein that is 1498 amino acids in length. Conversely, the skipping of exon 7 is represented at the cDNA and protein levels as c.114_216del p.Ser38Argfs*11. This event alters the reading frame, leading to a premature termination codon (PTC) within exon 8, which may result in a truncated protein of 47 amino acids. Collectively, these minigene results suggest that the mutation significantly impacts mRNA splicing, leading to the generation of abnormal proteins that may contribute to the patient's disease.

### 3.6 Prediction of the Impact of Aberrant Splicing on Protein Structure

Through in vivo splicing and minigene analysis, we identified the potential presence of proteins translated from aberrant mRNA within the patient's body. The truncated protein produced by the abnormal band c is considerably shorter than the normal protein and has lost several of its original functional domains. We hypothesize that this truncated protein may have consequently lost its original function. The following results are based on bioinformatics analysis techniques and computational predictions and represent potential future research directions.

**FIGURE 4:**
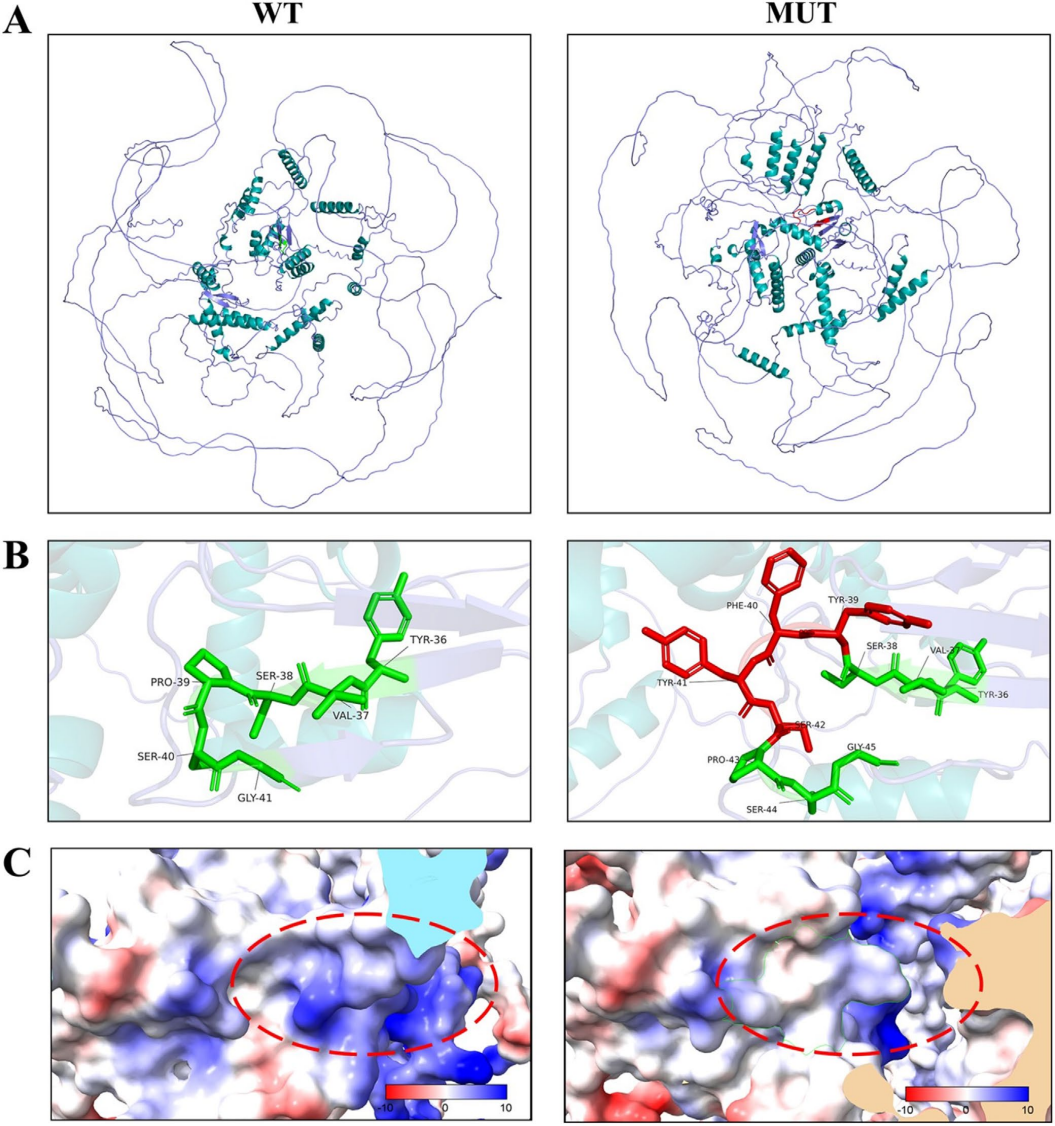
(A) The three‐dimensional spatial structure of the protein reveals notable differences in the folding of the amino acid sequence and α‐helices attributable to the mutation. The left side illustrates the wild‐type protein, while the right side depicts the mutant protein. (B) Upon magnifying the region surrounding the mutation site, the amino acids 36–41 of the wild‐type protein are displayed on the left (marked in green), whereas the four inserted amino acids (Tyr, Phe, Tyr, Ser) in the mutant protein are highlighted in red on the right, with the original amino acids also marked in green. Observations indicate changes in the spatial structure of the amino acid sequence, accompanied by interactions with neighboring amino acids that may influence the protein's main‐chain structure. (C) The surface electrostatic potential distribution map of the protein, generated using ChimeraX software, highlights the area of abnormal amino acid insertion within a circle. The wild‐type protein on the left exhibits a distinct positive electrostatic potential in the region of the mutation site, while the mutated protein on the right shows a significantly reduced electrostatic potential in this area, nearing electro‐neutrality. These alterations may impact the protein's interactions with other molecules. In the map, blue represents regions of positive charge distribution, red indicates areas of negative charge distribution, and white signifies non‐charged regions.

We conducted an analysis of the potential proteins that may be generated by band b after the mutation using bioinformatics prediction tools. Utilizing PyMOL and ChimeraX, we modeled the three‐dimensional structure and the electrostatic potential distribution on the surface of the aberrant protein, which contains an insertion of four amino acids. A detailed examination of the three‐dimensional spatial structure of the *MBD5* protein revealed significant alterations in its folding structure (Figure 4A). These structural modifications stem from the insertion of the four amino acids, which subsequently influence the overall conformation of the protein. Additionally, a localized magnification analysis provided insights into the binding interactions of the inserted amino acids (Tyr, Phe, Tyr, Ser) with the adjacent normal amino acids. The amino acid sequence of the wild‐type protein is indicated in green, while the inserted four amino acids are represented in red (Figure 4B). These inserted amino acids not only engage spatially with the surrounding amino acids but may also disrupt the main‐chain structural stability of the *MBD5* protein by altering the local chemical environment. Such structural disruptions could potentially affect the protein's functional domains, thereby influencing its biological functions.

To gain a more comprehensive understanding of the impact of the mutation on protein charge distribution, we employed the Adaptive Poisson‐Boltzmann Solver (APBS) within ChimeraX software to conduct a thorough analysis of the surface electrostatic potential distribution. Our prediction results show that, prior to the mutation, the protein exhibited a distinct positive electrostatic potential in the region surrounding the mutation site, suggesting that this area likely engages in significant charge interactions under normal conditions. In contrast, following the mutation, the electrostatic potential in this region was markedly diminished, approaching electro‐neutrality (Figure 4C). This alteration implies that the mutation not only affects the local structure of the protein but may also modify the distribution of the protein's surface electrostatic potential. Such changes in electrostatic potential could influence the protein's interactions with other molecules.

## 4 Discussion

Autosomal Dominant Intellectual Disability Type 1 (Mental Retardation, Autosomal Dominant 1, MBD1) is a rare neurodevelopmental genetic disorder characterized by developmental and language impairments, and it is closely associated with DNA methylation, making it a significant focus of research. Within the human genome, genes involved in DNA methylation primarily include the DNA methyltransferase family, the Ten‐eleven translocation (TET) protein family, and the MBD protein family. These genes and proteins work collectively to regulate gene expression and protein function through the process of DNA methylation. Mutations in these genes can have severe consequences. The *MBD5* protein, a member of the MBD protein family, is crucial to this process. The protein encoded by the *MBD5* gene contains two essential domains: an MBD domain, which is the minimal region required for MBD to specifically bind to methylated DNA, and a PWWP (Pro‐Trp‐Trp‐Pro) domain, which is important for the function of many proteins involved in cell proliferation and differentiation. Mutations in the *MBD5* gene are typically associated with neurodevelopmental disorders, traits of autism spectrum disorder, and cognitive impairments. The most prevalent mutation is *MBD5* haploinsufficiency, which is linked to syndromes that include microcephaly, intellectual disability, severe speech impairments, and epileptic seizures.

In this study, we experimentally demonstrated that the mutation c.114‐13A>G induces aberrant splicing of the *MBD5* gene, which alters the mRNA sequence and results in phenomena such as base retention and exon skipping. We hypothesize that these abnormal transcripts may lead to changes in the amino acid sequence, thereby producing dysfunctional proteins that impair the normal function of the *MBD5* protein, ultimately contributing to neurodevelopmental disorders in affected patients. This research has revised the ACMG classification of this mutation, providing significant experimental evidence for clinical practice and offering a methodological framework for investigating similar intronic mutations.

In parallel, we must closely examine the two abnormal transcripts identified in the patient. Through comprehensive experimentation and analysis of various samples, we performed in vivo splicing validation for the patient and their family members. Our findings indicate that the abnormal splicing transcripts in the patient are likely the primary contributors to the disease. These transcripts may induce alterations in the amino acid sequence of the protein and may even result in the premature emergence of a stop codon, thereby producing truncated proteins. Furthermore, these abnormal transcripts may be subjected to degradation prior to translation via the nonsense‐mediated mRNA decay (NMD) mechanism, which prevents the accumulation of abnormal proteins. However, this degradation process can lead to haploinsufficiency, a condition that is pathogenic.

Our research findings confirm that the mutation impacts mRNA splicing, suggesting the presence of abnormal proteins in the patient. Through 3D protein structure analysis and evaluation of protein surface electrostatic potential, we observed significant differences in the spatial structure of the mutated protein. Further validations will be performed at the protein level to investigate the functional structural differences between the mutated and normal proteins. We aim to explore the effects of *MBD5* gene mutations on DNA methylation, thereby elucidating the pathogenic molecular mechanisms associated with MBD1. Alternatively, we can analyze the degradation pathways of proteins expressed in vitro to experimentally demonstrate the potential degradation routes of the small quantities of abnormal *MBD5* proteins found in normal individuals. Additionally, we plan to develop targeted clinical treatment strategies to mitigate the overaccumulation of abnormal proteins within cells.

As of the current date, the Human Gene Mutation Database (HGMD) has documented 192 confirmed pathogenic mutations in the *MBD5* gene. These mutations are categorized as follows: 41 missense/nonsense mutations (21.3%), 8 splice site mutations (4.2%), 24 small deletions (12.5%), 4 small insertions (2.1%), 1 small indel (0.5%), and 114 chromosomal structural variations (59.4%). Excluding chromosomal structural variations, a total of 36 point mutations were identified in the cDNA sequence. Among these, mutations in exon 9 were the most frequent, accounting for 16 pathogenic mutations (44.4%), followed by exons 8 and 12, each with 5 mutations (13.9%). Exon 6 contained 3 mutations (8.3%), while exons 11 and intron 8 each had 2 mutations (5.5%). Exons 7, 10, and intron 11 each had 1 mutation (2.8%). The distribution of mutations is significantly concentrated in the exon 8–9 region, suggesting that this area may serve as a hotspot for pathogenic mutations. These mutations are likely to have a substantial negative impact on the function of the *MBD5* gene, thereby possessing considerable research value. Although intronic mutations represent a smaller proportion of pathogenic mutations, the de novo mutation (c.114‐13A>G) studied here is located in intron 6. An in‐depth analysis of this de novo mutation has revealed its potential pathogenicity. This discovery expands the known mutation spectrum of the *MBD5* gene and provides new experimental evidence for intronic splicing variants that lead to disease. It enriches the pathogenic sites cataloged in the HGMD database and offers critical reference information for future gene diagnosis of Mental Retardation, Autosomal Dominant 1.

In summary, our study enhances the understanding of the mutation spectrum of the *MBD5* gene and further substantiates the association between Autosomal Dominant Intellectual Disability Type 1 and the *MBD5* gene. Through both in vitro and in vivo experiments, we have demonstrated that this mutation disrupts mRNA splicing, resulting in the production of abnormal transcripts characterized by exon skipping and base retention, which consequently affect normal function. However, to comprehensively elucidate the molecular mechanisms underlying this hereditary disease, further investigation into the pathogenic mechanisms of the abnormal proteins associated with Autosomal Dominant Intellectual Disability Type 1 is essential. These findings may ultimately pave the way for novel diagnostic and therapeutic strategies for patients affected by Autosomal Dominant Intellectual Disability Type 1.

## Author Contributions

**Heng Jiang:** formal analysis, investigation, methodology, writing – original draft, writing – review and editing. **Jingjing Mou:** funding acquisition, methodology, resources, data curation, writing – review and editing. **Qiwei Zhao:** investigation, visualization, validation. **Long Ding:** investigation, software, methodology, writing – review and editing. **Yu Wang:** visualization, software, validation, writing – review and editing. **Xiaohong Guo:** data curation, software, visualization, project administration. **Guohua Yang:** conceptualization, data curation, resources, supervision, visualization, writing – review and editing.

## Conflicts of Interest

The authors declare no conflicts of interest.

## Supporting information


Data S1.

## Data Availability

The data that support the findings of this study are openly available in MBD5 DATA at https://data.mendeley.com/preview/pn67m7ttf3?a=a6a5968b‐4279‐41bb‐8fa1‐d7954be3a58a. doi: https://doi.org/10.17632/pn67m7ttf3.2.
