# Supplementary material for: A Novel Intronic Mutation in MBD5 Results in Autosomal Dominant Intellectual Disability Type 1 due to Abnormal Splicing
Source: Mol Genet Genomic Med. 2025 Jul 15;13(7):e70121. doi: 10.1002/mgg3.70121 (PMC12261026; doi:10.1002/mgg3.70121)
Supplement: Supplementary file 1 — Data S1. [file MGG3-13-e70121-s001.zip › MGG370121-sup-0001-Supplementary Materials_1.docx]

**Analysis of the pcMINI-C Results for the MBD5 Gene**

In the pcMINI-C vector, a segment comprising part of intron 6 (641 bp) - exon 7 (103 bp) - intron 7 (1054 bp) - exon 8 (181 bp) was inserted into the pcMINI-C vector (Fig. 1A). After transfection, the splicing pattern of exon A - exon 7 - exon 8 was observed to determine if any abnormalities existed. The results are shown in Figure 1. In both HeLa and 293T cells, the wild-type MBD5 gene transcript appeared as a single band, consistent with the expected size of 546 bp, designated as band a. This band was purified from both cell lines and directly subjected to Sanger sequencing. In HeLa and 293T cells, the mutant type appeared as two bands, with the larger band designated as band b and the smaller band as band c (Fig. 3B). The mutant bands from both cell lines were purified, TA cloned, and then subjected to Sanger sequencing. The results showed that the wild-type band a was a normally spliced band, with a splicing pattern of exon A (192 bp) - exon 7 (103 bp) - exon 8 (181 bp). The mutant band b was an abnormally spliced band, with a 12 bp retention on the right side of intron 6, and a splicing pattern of exon A (192 bp) - ▽intron 6 (12 bp) - exon 7 (103 bp) - exon 8 (181 bp). The mutant band c was an abnormally spliced band, with exon 7 skipping, and a splicing pattern of exon A (192 bp) - exon 8 (181 bp) (Fig. 1D).


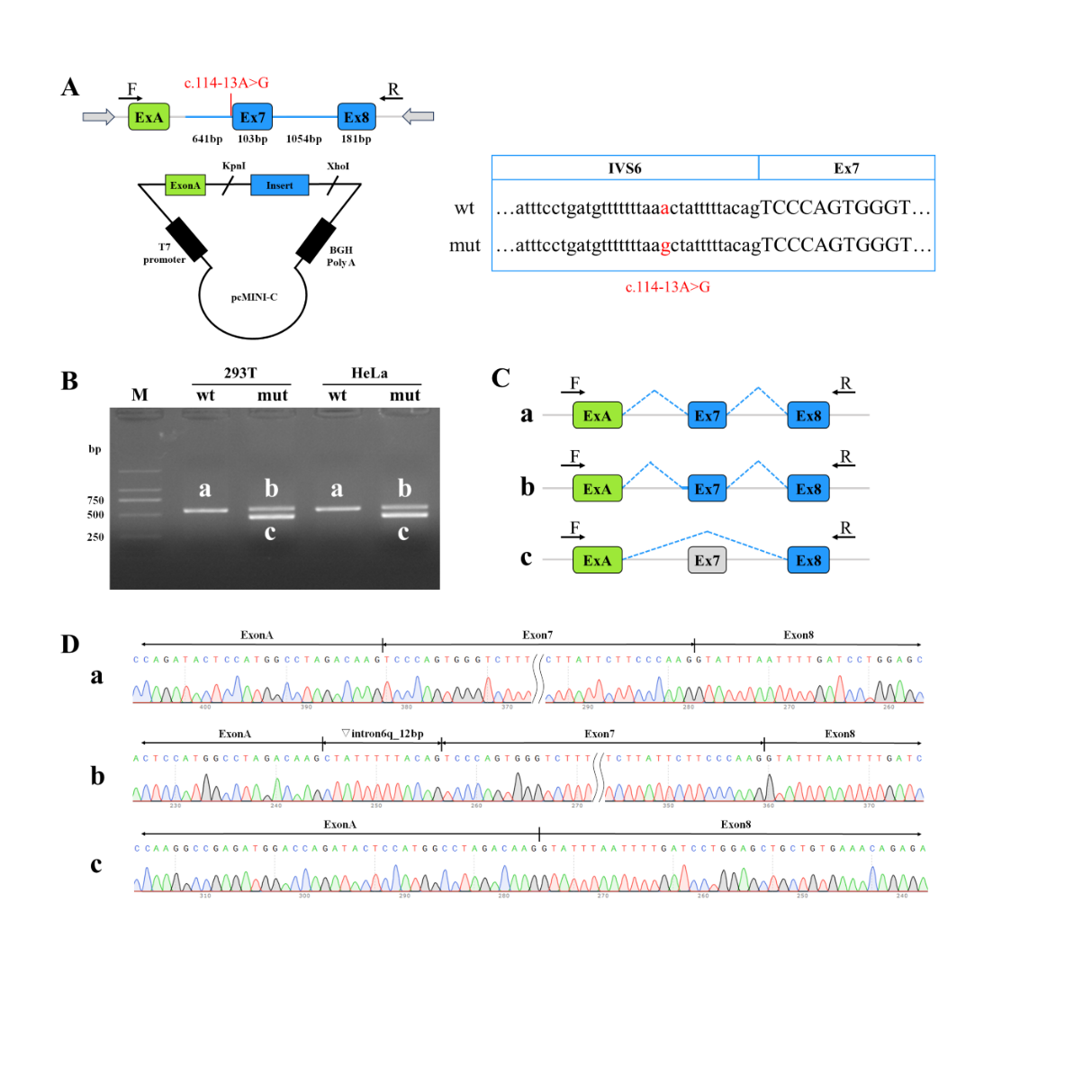


Fig.1 The results of the pcMINI-c vector for the MBD5 gene. A. Schematic diagram of the pcMINI-c vector construction; B. Agarose gel electrophoresis of RT-PCR transcription analysis; bands labeled as a, b, and c in HeLa and 293T cells; C. Schematic of splicing for different bands; D. Sanger sequencing results for different bands.
