# Supplementary material for: A Novel Intronic Mutation in MBD5 Results in Autosomal Dominant Intellectual Disability Type 1 due to Abnormal Splicing
Source: Mol Genet Genomic Med. 2025 Jul 15;13(7):e70121. doi: 10.1002/mgg3.70121 (PMC12261026; doi:10.1002/mgg3.70121)
Supplement: Supplementary file 1 — Data S1. [file MGG3-13-e70121-s001.zip › MGG370121-sup-0002-Supplementary Materials_2 Table 1_PCR primer sequences.docx]

**Table 1: PCR primer sequences**

| PCR Primer Names | PCR primer base sequences (5′-3′) |
| --- | --- |
| 2530-MBD5-F | gcagtggttgaactcgtcac |
| 2811-MBD5-F | atgaacttggtcagagggct |
| 5490-MBD5-R | ccccaggaaaaaggctacat |
| 5669-MBD5-R | cttgcaacttttctgcagct |
| MBD5-mut-F | atttcctgatgtttttttaaGctatttttacagTCCCAGTG |
| MBD5-mut-R | CACTGGGActgtaaaaatagCttaaaaaaacatcaggaaat |
| pcMINI-MBD5-KpnI-F | ggtaGGTACCcttactgacttttgtgatct |
| pcMINI-MBD5-XhoI-R | tttcCTCGAGccttgcaaatgagtaaaaca |
| pcMINI-C-MBD5-KpnI-F | ggtaGGTACCttgtcaactctgcttaattc |
| pcMINI-C-MBD5-XhoI-R | tagactcgAGATTTGTTCCTCCTCCGGGACTG |
| pcMINI-F | ACTTAAGCTTatgagtgggctttggggtggccggtt |
| pcMINI-R | TAGAAGGCACAGTCGAGG |
| pcMINI-C-F | ACTTAAGCTTatgagtgggctttggggtggccggtt |
| pcMINI-C-R | TAGAAGGCACAGTCGAGG |
| MBD5-F1 | ACTTGGTTCCAAACTGAGCT |
| MBD5-F2 | GGAGAGTCCCTAGCAGACAC |
| MBD5-R1 | TTGGAGTTGCTGCCCGAGAA |
| MBD5-R2 | CAGGGTGGAGTTCTTGTTGT |
