# Supplementary material for: A Novel Intronic Mutation in MBD5 Results in Autosomal Dominant Intellectual Disability Type 1 due to Abnormal Splicing
Source: Mol Genet Genomic Med. 2025 Jul 15;13(7):e70121. doi: 10.1002/mgg3.70121 (PMC12261026; doi:10.1002/mgg3.70121)

Supplementary Material 3

The list of all candidate variants identified during the whole-genome sequencing (WGS) process of the patient.


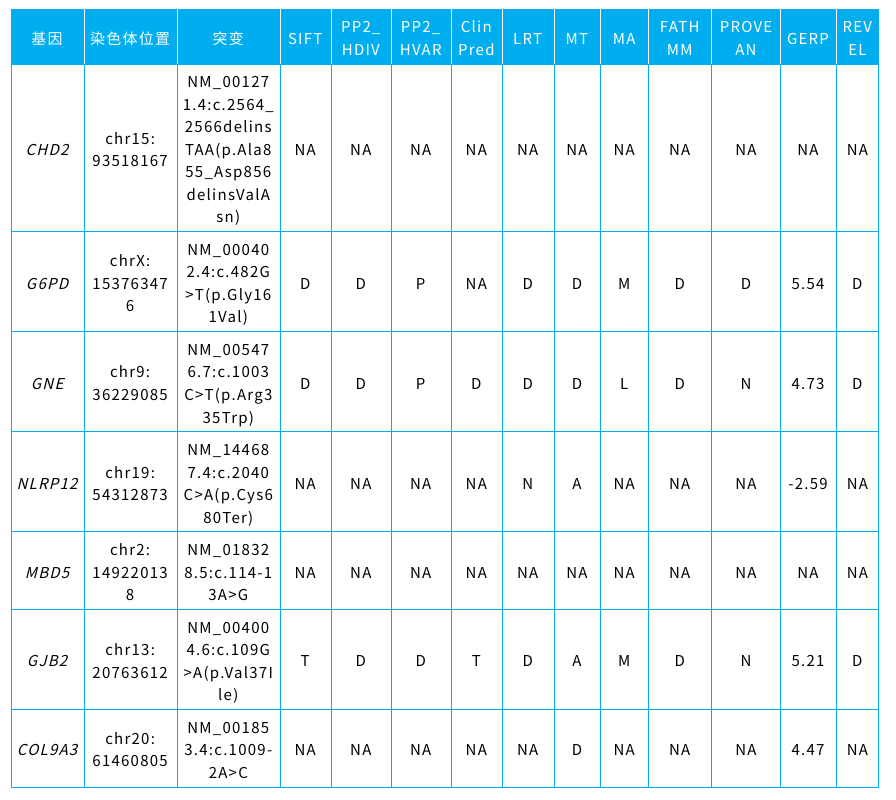


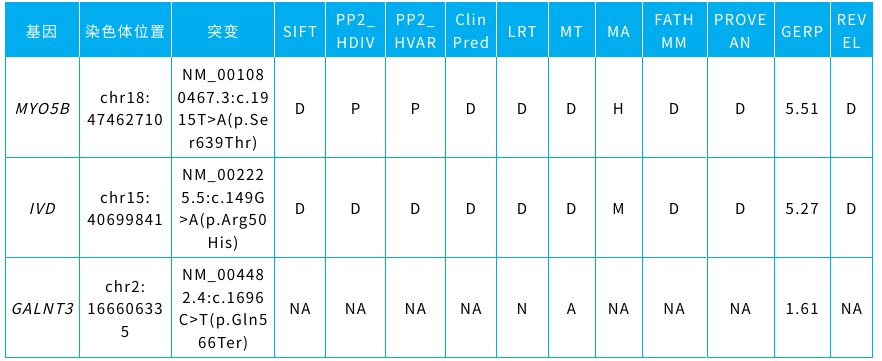


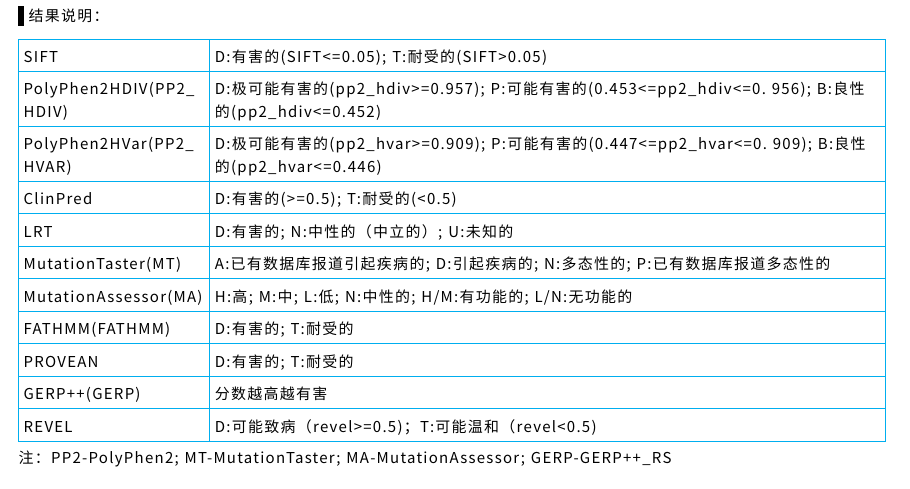

Supplement: Supplementary file 1 — Data S1. [file MGG3-13-e70121-s001.zip › MGG370121-sup-0003-Supplementary Materials_3.docx]
