# Supplementary material for: A Novel Intronic Mutation in MBD5 Results in Autosomal Dominant Intellectual Disability Type 1 due to Abnormal Splicing
Source: Mol Genet Genomic Med. 2025 Jul 15;13(7):e70121. doi: 10.1002/mgg3.70121 (PMC12261026; doi:10.1002/mgg3.70121)

Supplementary Material 4

Schematic diagram of the mutation position in intron 6 and exon 7

The c.114-13A>G mutation in the MBD5 gene is located at the -13 position of Intron 6, where the base at position c.114-13 changes from A to G. The corresponding position near the 6th intron and the 7th exon is shown in the figure.

Intron6(3710bp) Exon7(103bp) Intron7(1054bp)


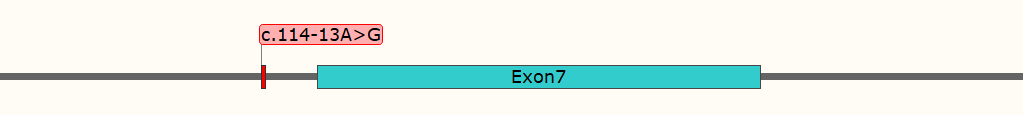


Functional prediction of the MBD5 c.114-13A>G mutation

Prediction 1 — SpliceAI: The algorithm indicates that the original acceptor site is lost after the mutation, with a confidence score decrease of 0.68. A new acceptor site is generated near the mutation site, with a confidence score of 0.18, suggesting that the mutation affects splicing.


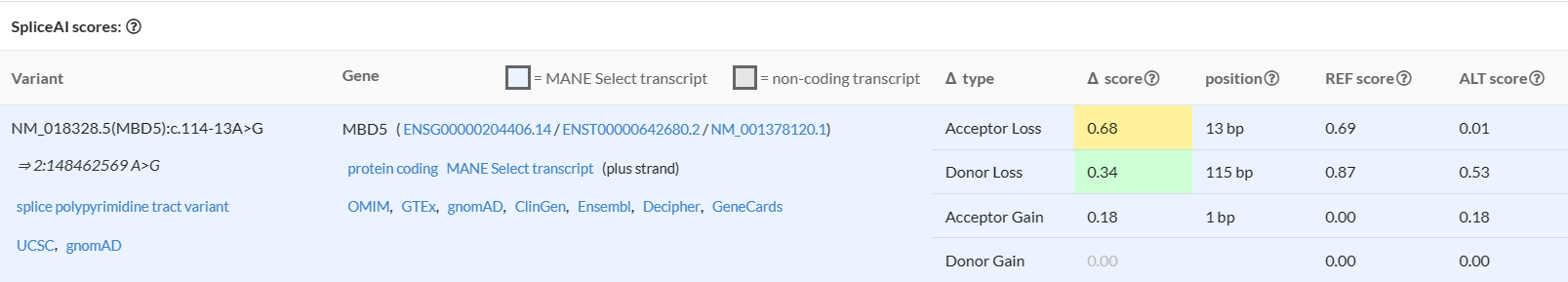


Prediction 2 — BDGP: The algorithm indicates that a new acceptor site is generated near the mutation site after the mutation, suggesting that the mutation affects splicing.

Before Mutation After Mutation


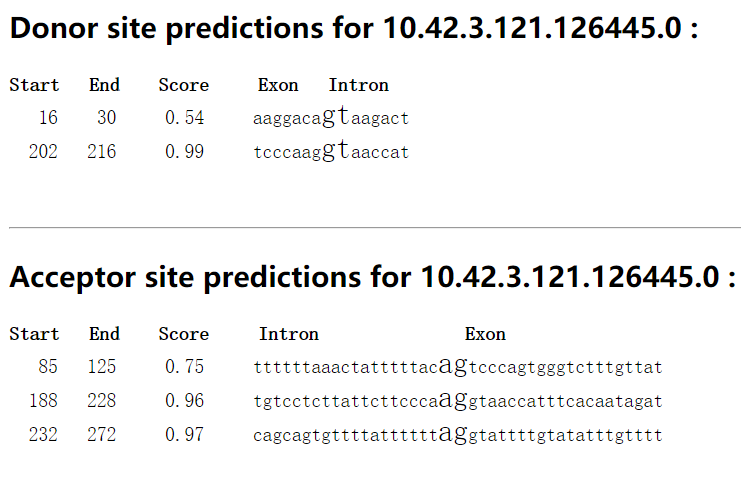

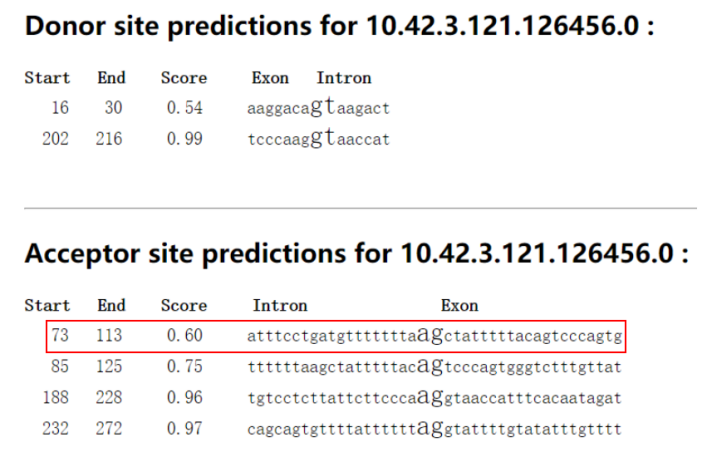


Prediction 3 — RDDC^SC^: The algorithm indicates that after the mutation, intron retention or splicing consistent with the wild type or exon skipping may occur, suggesting that the mutation may affect splicing.


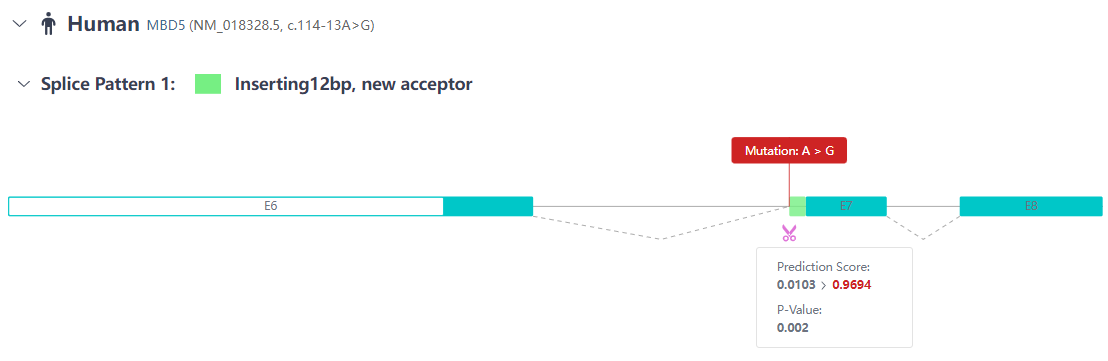

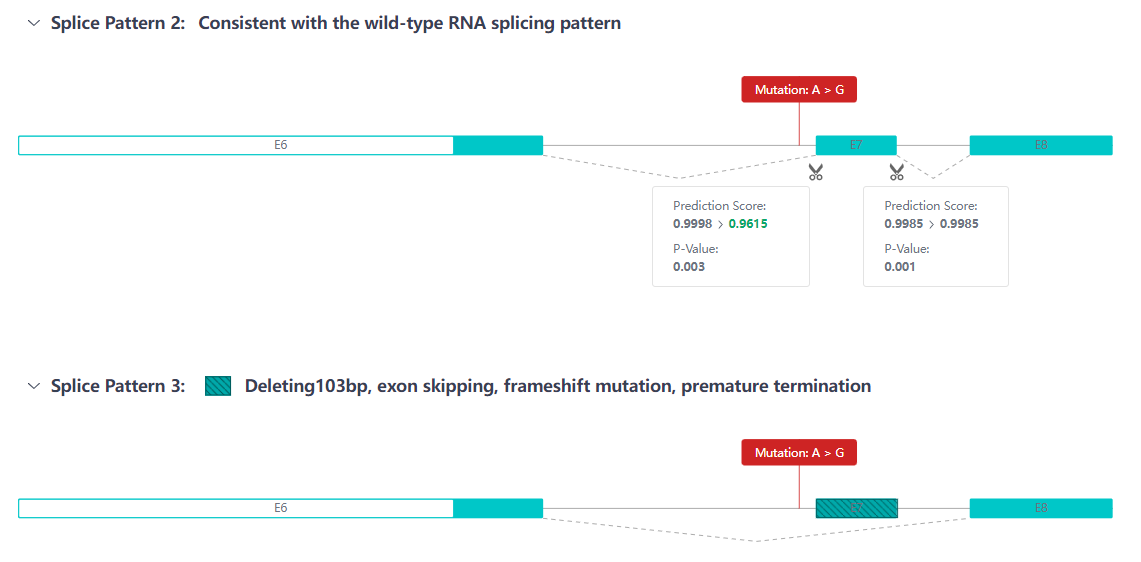

Supplement: Supplementary file 1 — Data S1. [file MGG3-13-e70121-s001.zip › MGG370121-sup-0004-Supplementary Materials_4.docx]
